# Shock wave-induced ATP release from osteosarcoma U2OS cells promotes cellular uptake and cytotoxicity of methotrexate

**DOI:** 10.1186/s13046-016-0437-5

**Published:** 2016-10-03

**Authors:** Baochang Qi, Tiecheng Yu, Chengxue Wang, Tiejun Wang, Jihang Yao, Xiaomeng Zhang, Pengfei Deng, Yongning Xia, Wolfgang G. Junger, Dahui Sun

**Affiliations:** 1 Division of Orthopedic Traumatology, The First Hospital of Jilin University, NO.71 Xinmin Street, Changchun, 130021 China; 2 Department of Surgery Beth Israel Deaconess Medical Center, Harvard Medical School, Boston, MA USA; 3 Ludwig Boltzmann Institute for Traumatology, Vienna, A-1200 Austria

**Keywords:** Membrane permeabilization, Human osteosarcoma, Extracorporeal shock waves, Methotrexate, P2X7 purinergic receptors

## Abstract

**Background:**

Osteosarcoma is the most prevalent primary malignant bone tumor, but treatment is difficult and prognosis remains poor. Recently, large-dose chemotherapy has been shown to improve outcome but this approach can cause many side effects. Minimizing the dose of chemotherapeutic drugs and optimizing their curative effects is a current goal in the management of osteosarcoma patients.

**Methods:**

In our study, trypan blue dye exclusion assay was performed to investigate the optimal conditions for the sensitization of osteosarcoma U2OS cells. Cellular uptake of the fluorophores Lucifer Yellow CH dilithium salt and Calcein was measured by qualitative and quantitative methods. Human MTX ELISA Kit and MTT assay were used to assess the outcome for osteosarcoma U2OS cells in the present of shock wave and methotrexate. To explore the mechanism, P2X7 receptor in U2OS cells was detected by immunofluorescence and the extracellular ATP levels was detected by ATP assay kit. All data were analyzed using SPSS17.0 statistical software. Comparisons were made with *t* test between two groups.

**Results:**

Treatment of human osteosarcoma U2OS cells with up to 450 shock wave pulses at 7 kV or up to 200 shock wave pulses at 14 kV had little effect on cell viability. However, this shock wave treatment significantly promoted the uptake of Calcein and Lucifer Yellow CH by osteosarcoma U2OS cells. Importantly, shock wave treatment also significantly enhanced the uptake of the chemotherapy drug methotrexate and increased the rate of methotrexate-induced apoptosis. We found that shock wave treatment increased the extracellular concentration of ATP and that KN62, an inhibitor of P2X7 receptor reduced the capacity methotrexate-induced apoptosis.

**Conclusions:**

Our results suggest that shock wave treatment promotes methotrexate-induced apoptosis by altering cell membrane permeability in a P2X7 receptor-dependent manner. Shock wave treatment may thus represent a possible adjuvant therapy for osteosarcoma.

## Background

Osteosarcoma is the most prevalent primary malignant bone tumor affecting both adults and children [1] and accounts for 2.4 % of all pediatric cancers [2]. Osteosarcoma arises from osteoid tissues [3] and is currently treated with chemotherapy and surgery [4]. Despite significant improvements in the treatment of osteosarcoma in recent decades, the 5-year survival rate for osteosarcoma is still only not more than 60 % [5]. Alternative treatments have also been employed [6, 7]. For example, Wang et al. reported triazine-modified dendrimer for efficient TRAIL gene therapy in osteosarcoma. In their study, a triazine-modified dendrimer G5-DAT 66 was synthesized and used as a vector for TRAIL gene therapy in vitro and in vivo, and their results suggested that triazine-modified dendrimer has promising potential for TRAIL gene therapy in osteosarcoma [7]; but these treatments achieved only marginal improvements in comparison to conventional chemotherapy. Therefore, conventional chemotherapy is still the predominant treatment for osteosarcoma. However, high doses of cytotoxic chemotherapy can cause life-threatening side effects, including cardiotoxicity and nephrotoxicity. Balancing the dose of chemotherapeutic drugs to reduce side effects and maintain the curative efficacy of chemotherapeutics remains a challenge in the care for osteosarcoma patients.

Recent advances suggest that shock wave treatment may sensitize osteosarcoma cells to chemotherapeutic drugs [8]. Shock waves are elicited by a transient pressure disturbance and characterized by high positive pressure. The rapid pressure transition of shock waves exerts very high tension on exposed surfaces, damaging their structure [9, 10]. Approximately 20 years ago, extracorporeal shock waves were introduced for the treatment of kidney stones and have substantially improved the treatment of urolithiasis [11, 12]. Subsequently shock wave therapy has been adopted for orthopedics and traumatology to treat various insertional tendinopathies (enthesiopathies) with delayed unions and nonunions of fractures and for the treatment of various musculoskeletal diseases [11, 12]. Shock waves were also reported to enhance the cytotoxicity of doxorubicin and methotrexate in osteosarcoma cell lines [8], suggesting that shock waves may sensitize osteosarcoma cells to conventional chemotherapeutics. However, the mechanisms underlying this effect remained unknown.

In this study, we treated human osteosarcoma U2OS cells with methotrexate (MTX) in the presence or absence of extracorporeal shock waves and assessed cell viability and membrane permeability. We found that shock waves enhanced the cytotoxic effect of MTX by increasing cell membrane permeability through the secretion of ATP and stimulation of P2X7-type ATP receptors of osteosarcoma U2OS cells.

## Methods

### Cell culture

Human osteosarcoma U2OS cells were obtained from the Shanghai Institute of Cell Biology (Cell Bank, Chinese Academy of Sciences, Shanghai, China) and cultured in Roswell Park Memorial Institute (RPMI)-1640 medium (HyClone, Beijing, China) supplemented with 1 % penicillin-streptomycin (HyClone, Beijing, China), 10 % (vol/vol) fetal bovine serum (HyClone, Beijing, China) in 250 ml culture flasks at 37 °C in an humidified atmosphere of 5 % CO_2_. Once every 4 days, cells were detached with trypsin/EDTA (HyClone, Beijing, China), counted and re-seeded at 1 × 10^6^ cells/flask.

### Extracorporeal shock waves exposure

Using a model KDE-2001 extracorporeal shock wave lithotripter (Beijing Zhongke Jian’an Meditechnics, Beijing, China) shock waves were generated by underwater spark discharge from an electrode jacketed with an ultraviolet light (UV)-resistant outer membrane. Cells were transferred to 1.8 ml polypropylene tubes. The propagation waves were focused on the center of the liquid column, located by X-ray. Test tubes were wrapped in a triple layer of Parafilm and stored in an ice bath before and after application of shock wave. Using a generator, 7 kV or 14 kV shock waves were generated with a capacitance of 0.5 μF and a frequency of 50 Hz. Cells (1 × 10^6^ cells/ml in 0.25 ml) in a 1.8 ml polypropylene round-bottom tube were exposed to 0, 50, 100, 150, 200, 250, 300, 350, 400, 450, 500, 1,000, or 1,500 shock wave pulses at 7 kV or 14 kV. The numbers viable cells were determined by trypan blue dye exclusion assay using a hemocytometer. Each condition was repeated in triplicate in three independent experiments.

### Fluorophores

Cellular uptake of the fluorophores Lucifer Yellow CH dilithium salt (457.25 Da, L0259, λex 428 nm, λem 540 nm) and Calcein (622.53 Da, C0875, λex 470 nm, λem 509 nm), acquired from Sigma (Saint Louis, MO, USA), was measured. A Lucifer Yellow CH dilithium salt (LY) solution (1 mM) was prepared in phosphate-buffered saline (PBS) without Mg^2+^ and Ca^2+^. A Calcein solution (0.2 mM) was prepared in 1 M NaOH. Living and damaged cells were distinguished by propidium iodide (PI) staining. PI (20 μg/ml) was acquired from Beyotime (Haimen, China) and was added to the cells for 5 min before capturing fluorescence images under a fluorescence microscope (λex 535 nm, λem 615 nm; Olympus, IX51) at 20 °C. An excitation of 428 nm was used for LY and 470 nm for Calcein. Extracellular fluorophores were removed by washing 3 times with PBS, and samples were resuspended in PBS. For qualitative detection, cells were added to 12-well plates preconditioned with fibronectin (F8180; Beijing Solarbio Science & Technology, Beijing, China) to aid attachment. After 30 min, cells were observed under a microscope. For quantitative determination, cells were resuspended in 1 ml PBS and sonicated for 1 min (Model VC × 750, Sonics & Materials). Then 100 μl of sonicated material was added to a 96-well black bottom plate and fluorescence was read at 20 °C with a Thermo Scientific Fluoroskan Ascent FL at an excitation of 430 nm and an emission of 540 nm for LY, and an excitation of 460 nm and an emission of 510 nm for Calcein.

### MTX assay

MTX was dissolved in DMSO and tri-distilled water and cells were divided into 4 groups: control group, MTX group (100 ng/ml MTX), MTX + S1 group (100 ng/ml MTX 400 7 kV shock waves), MTX + S2 group (100 ng/ml MTX, 150 14 kV shock waves). A sample of cells (1 × 10^6^) from each condition was washed with PBS three times and intracellular MTX was quantified by Human MTX ELISA Kit (Shanghai Heng Yuan Biological Technology, Shanghai, China). The kit uses a biotinylated sandwich enzyme-linked immunosorbent assay to assess MTX levels in samples. Briefly, MTX was added to the well pre-coated with anti-MTX monoclonal antibody, followed by sequential incubation and addtion of biotinylated anti-MTX antibody, HRP conjugated streptavidin-biotin and DAB substrate. The remaining cells (1 × 10^6^) were cultured for 24 h and viability was assessed by MTT assay.

### Detection of P2X7 in U2OS cells by immunofluorescence

U2OS cells were resuspended in 4 % paraformaldehyde solution to a density of 1 × 10^6^ cells/ml. Then 5 μl of cell suspension was dropped on Superfrost plus slides and incubated at 50 °C for 15 min, followed by incubation at 37 °C for 24 h with 2 μl P2RX7 polyclonal antibody (11144-1-AP, 1:20, Proteintech, Chicago, IL, USA) in a humidified atmosphere with 5 % CO_2._ Slides were washed three times with PBS, then incubated at 50 °C for 15 min, followed by incubation for 1 h at 37 °C with 2 μl TRITC-conjugated goat anti-rabbit IgG (H + L) secondary antibody (SA00007-2, 1:20, Proteintech) in a humidified atmosphere with 5 % CO_2_. The slides were washed in tri-distilled water and observed under fluorescence microscope. The control group was incubated without primary antibody.

### Detection of extracellular ATP levels

Cells at 70–80 % confluency were resuspended to 2 × 10^6^ cells/ml, and 0.25 ml of cell suspension was added to 1.8 ml of Eppendorf tubes and treated with 400 rounds of shock waves at 7 kV or 150 rounds at 14 kV in the presence or absence of GdCl_3_ to block mechanically stimulated ATP channels [13] or Brefeldin A (BFA) to inhibit vesicular ATP release [13]. Extracellular ATP concentrations were determined using an ATP assay kit (Shanghai Heng Yuan Biological Technology) according to the manufacturer’s instructions.

### MTT assay

Cell viability was determined with a 3-(4, 5-dimethylthiazol-2-yl)-2, 5-diphenyltetrazolium bromide (MTT) assay. Cells at 70–80 % confluency were resuspended at 2 × 10^6^ cells/ml. Then 100 μl of cell suspension was added to each well of a 96-well plate and incubated with ATP (1, 10, 100, 500, or 1000 μM) for 24 h and 20 μl of 5 mg/ml MTT solution was added to each well and cells cultured for an additional 4 h. The culture medium was removed and 150 μl dimethylsulfoxide (DMSO) was added to dissolve formazan. Cell viability was quantified by measuring absorbance at 490 nm using a microplate spectrophotometer to calculate the optical density (OD) values.

### Annexin V-FITC/PI double staining of apoptosis

Cells of 70–80 % confluency were resuspended at 2 × 10^6^ cells/ml, and 0.25 ml of cell suspension was added to a 1.8 ml Eppendorf tube and treated with MTX and exposed to 400 shock wave pulses at 7 kV or 150 shock wave pulses at 14 kV in the presence or absence of the P2X7 receptor inhibitor KN62. Then, cell apoptosis was determined using an AnnexinV-FITC/PI apoptosis detection kit (Vazyme Biotech, Nanjing, China) according to the manufacturer’s instructions.

### Statistical analysis

All data were analyzed using SPSS17.0 statistical software and expressed as mean ± SD. Comparisons were made with *t* test between two groups. Group control data were used as the baseline for statistical comparison with other groups. A *P* value of <0.05 was considered statistically significant.

## Results

### Effect of shock wave treatment on U2OS cell viability

**Fig. 1:**
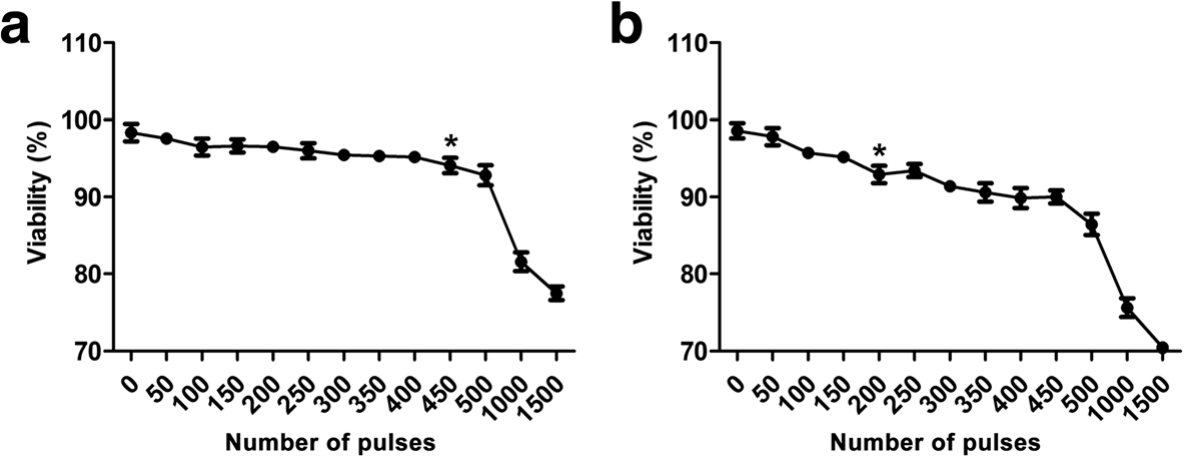
Determination of the optimal experimental conditions of shock waves for human osteosarcoma U2OS cells. U2OS cells were treated with the indicated number of shock at the voltage of 7 kV (**a**) or 14 kV (**b**). The cell viability was assessed by trypan blue exclusion assay. Data are mean ± SD, *n* = 5, *p < 0.05, Student^,^s *t*-test

To investigate optimal conditions for the sensitization of osteosarcoma cells to chemotherapy, human osteosarcoma U2OS cells were treated with 0, 50, 100, 150, 200, 250, 300, 350, 400, 450, 500, 1,000 or 1,500 shock wave pulses at 7 kV or 14 kV. Cell viability was assessed by trypan blue dye exclusion. We found that viability of U2OS cells remained >95 % following <450 shock wave pulses at 7 kV (Fig. 1a) or <200 pulses at 14 kV (Fig. 1b). Therefore, we subjected cells in subsequent experiments to 400 shock wave pulses at 7 kV or 150 pulses at 14 kV. We also treated MC3T3 cells, an osteoblast precursor cell line with these parameters and found that the viability of MC3T3 cells also remained >95 %, suggesting that this treatment does not harm normal bone cells.

### Shock waves stimulate uptake of Calcein and LY (Lucifer Yellow) by U2OS cells

**Fig. 2:**
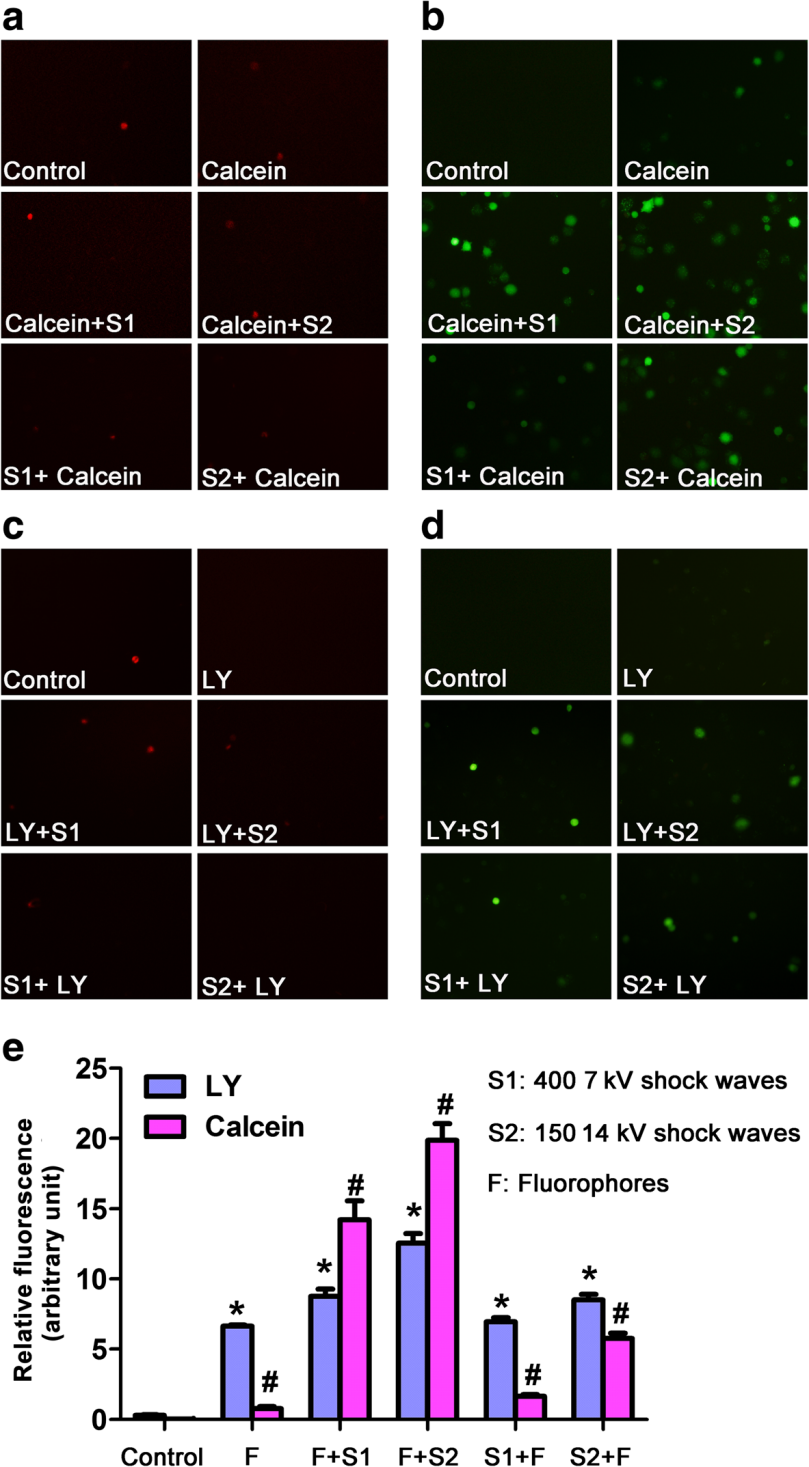
Shock waves enhanced the uptake of Calcein and LY in U2OS cells. **a**-**b**, U2OS cells were stained with Calcein (green) or PI (red) and treated with shock waves as described. **c**-**d**, U2OS cells were treated with LY and subjected to shock waves as indicated. LY (green) or PI (red) influx was measured. **e** Cells were treated as described and LY and Calcein influx was assessed. Data are mens ± SD, *n* = 3, *p < 0.05,Student^’^s *t*-test. **In calcein + S1: addition of calcein prior to shock wave treatment; in S1 + calcein: shock wave treatment prior to addition of calcein. Same for LY

To determine the effects of shock wave treatment on cell membrane permeability, U2OS cells were treated with shock waves as described above in the presence of LY or Calcein and cellular uptake of LY or Calcein was assessed by fluorescence microscopy. PI was used to distinguish between living and dead cells. We found that neither condition increased the proportion of dead cells (Fig. 2a and c). Cells treated with Calcein in combination with shock waves exhibited increased green fluorescence when compared to cells that were incubated in the absence of fluorophore or shock waves, or cells incubated with Calcein alone (Fig. 2b). Similarly, when compared to cells incubated in the absence of fluorophore or shock waves or to cells incubated with LY alone, cells treated with LY in combination with shock waves exhibited increased green fluorescence (Fig. 2d). Quantification of fluorescence intensity revealed that cells treated with Calcein followed by 400 shock wave pulses at 7 kV or 150 shock wave pulses at 14 kV exhibited highest fluorescence intensity (Fig. 2e) with statistical significance compared to untreated control (*P* < 0.05). These results indicate that shock waves promote cellular uptake of LY and Calcein with better effects by adding fluorophore prior to shock waves.

### Shock waves promote uptake of MTX and enhance its cytotoxicity on U2OS cells

**Fig. 3:**
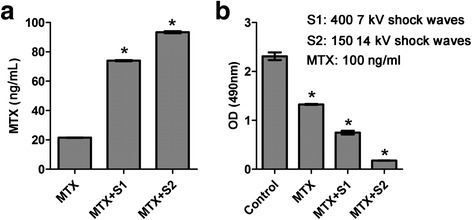
Shock waves significantly stimulated U2OS cell uptake of chemotherapy drug MTX and enhanced MTX cytotoxicity. **a** U2OS cells were treated with 100 ng/ml MTX followed by shock waves of 400 7 kV or 150 14 kV shock waves. Intracellular MTX concentrations were determined by Human MTX ELISA. **b** U2OS cells were treated with 100 ng/ml MTX followed by shock waves of voltage of 400 7 kV or 14 kV/150. The cells were cultured for 24 h and cell proliferation was tested by MTT assay. Data are mean ± SD, *n* = 3, *p < 0.05, Student^,^s *t*-test

Based on the above findings, we explored whether shock waves can promote cellular uptake of the chemotherapy drug MTX. We incubated U2OS cells with 100 ng/ml MTX followed by 400 shock wave pulses at 7 kV or 150 pulses at 14 kV. Then we determined intracellular MTX concentrations by using a human MTX ELISA assay. Both shock wave treatments significantly enhanced MTX uptake with greatest MTX uptake elicited by 150 shock wave pulses at 14 kV (*P* < 0.05, Fig. 3a). After treatment, the cells were cultured for 24 h and proliferation was assessed using an MTT assay. Compared to untreated controls, cells treated with MTX showed reduced viability, which was further reduced by shock wave treatment (*P* < 0.05, Fig. 3b). These results demonstrate that shock wave treatment significantly enhances the uptake of chemotherapy drug MTX and the cytotoxic effect of this anti-cancer drug on U2OS cells.

### Shock wave treatment of U2OS cells increases extracellular ATP concentrations

**Fig. 4:**
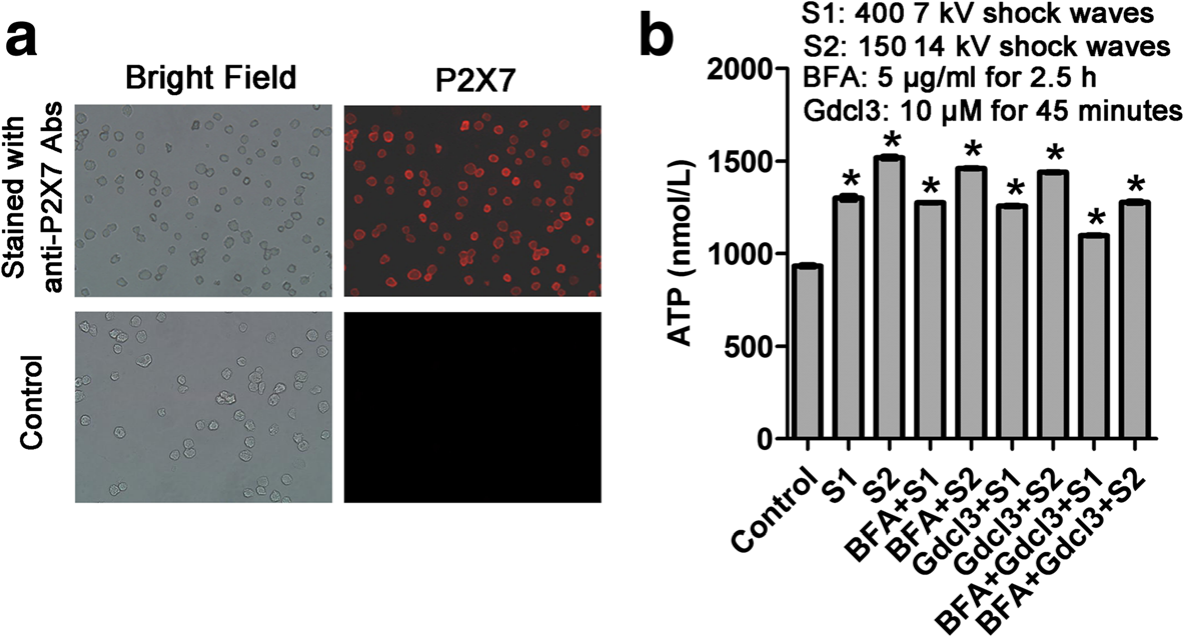
P2X7 was highly expressed on the cell surface and shock waves increased the extracellular concentration of ATP in U2OS cells. **a** P2X7 receptor expression in U2OS cells. Representative images of anti-P2X7 antibody separate experiments are shown. **b** Cells treated with BFA, GdCl_3_, and shock waves, and extracellular ATP concentrations were assessed in supernatants of the treated cells as indicated. Data are mean ± SD, *n* = 3, *p <0.05, Student, s *t*-test

To explore the mechanism underlying the enhancement of MTX uptake by shock wave treatment, we investigated whether ATP release is involved. First, we determined whether P2X7 receptors are present on the cell surface of cells using P2X7-specific antibodies. Using immune fluorescence staining, we found strong expression of P2X7 receptors on the surface of U2OS cells (Fig. 4a).

To assess whether shock waves promote ATP release, U2OS cells were treated with shock wave treatment as described above in the presence or absence of the ATP release inhibitors GdCl_3_ or Brefeldin A (BFA). Extracellular ATP concentrations were determined using ATP assay. Both shock wave treatments significantly increased extracellular ATP concentrations (*P* < 0.05). Moreover, GdCl_3_ and BFA alone or in combination reduced shock wave-induce release of ATP (Fig. 4b). Although the combination of GdCl_3_ and BFA reduced extracellular ATP concentrations, the inhibition was not significant or complete. This indicates that shock wave mediated ATP release involves mechanosensitive channels, vesicular mechanisms, and additional release most likely due to a third mechanism: the high-strength instant positive phase pressure that is generated by shock wave during a few seconds may cause a transient increase of cell membrane permeability, leading to a burst of ATP outflow.

### P2X7 receptor stimulation promotes cell permeability, MTX uptake, and cytotoxicity of U2OS cells

**Fig. 5:**
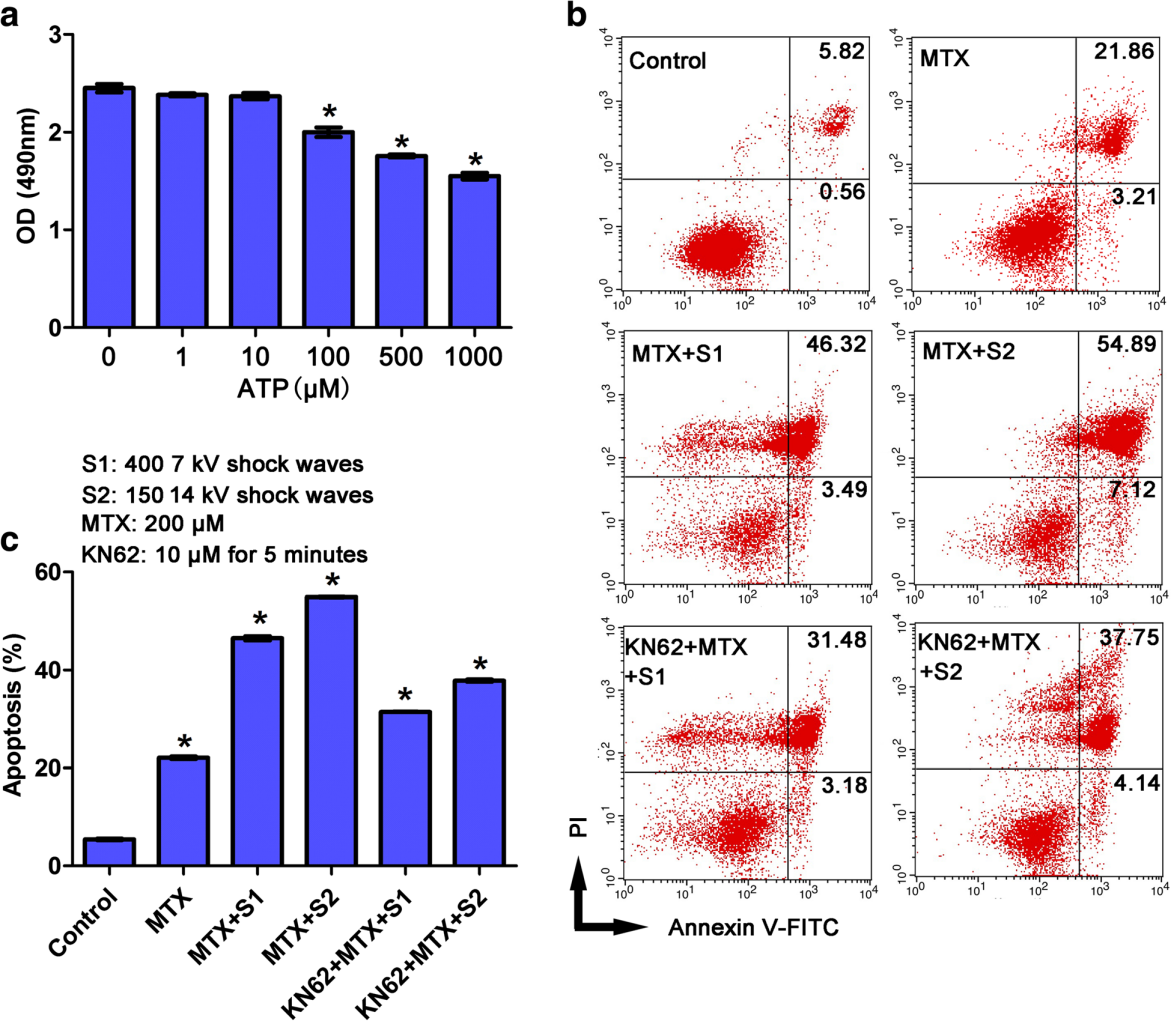
Alteration of cell membrane permeability by ATP/P2X7 contributed to the promotion of cell apoptosis of MTX by shock waves. **a** U2OS cells were treated with the indicated concentrations of ATP and cell viability was assessed by MTT assay. **b** U2OS cells were treated with MTX, shock waves and KN62 as indicated and apoptosis was assessed. Shown are representative histograms. **c** Quantitation of the apoptosis rates. Data are mean ± SD, *n* = 3, *p < 0.05,Student^,^s *t*-test

**Fig. 6:**
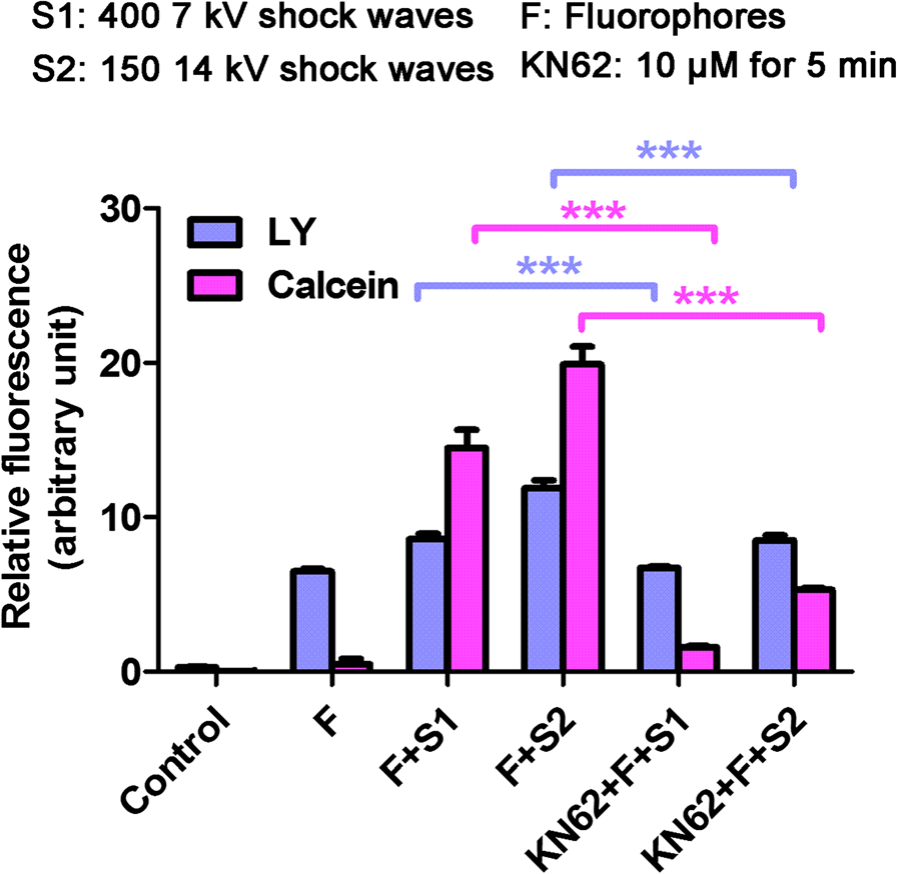
Shock waves significantly stimulated U2OS cell uptake of Calcein and LY. The uptake of Calcein and LY following addition of KN62 (10 μM for 5 min) were determined and significantly reduced intake of Calcein and LY following addition of KN62 was observed. Data are mean ± SD, *n* = 3, ***p < 0.05, Student^’^s *t*-test

We sought to determine whether shock waves promote MTX uptake and cytotoxicity by altering membrane permeability or by causing apoptosis due to the massive release ATP and stimulation of P2X7 receptors. We first treated U2OS cells with ATP and assessed cell viability using the MTT assay. As shown in Fig. 5a, ATP at concentrations <100 μM did not noticeably affect cell viability. However, ATP concentrations ≥100 μM significantly reduced cell viability (*P* < 0.05). Considering that shock wave treatment induced extracellular ATP concentrations of only 1.6 μM (Fig. 4b), these results suggest that ATP release does not contribute to shock wave-induced cell death by triggering P2X7 receptor-mediated apoptosis. Instead, these findings suggest that shock wave treatment opens cell membrane channels that facilitate MTX uptake. To test this hypothesis, we treated U2OS cells with MTX in the presence or absence of the P2X7 receptor inhibitor KN62 and we assessed apoptosis following shock wave treatment. We found that MTX-induced apoptosis was significantly weakened by KN62 treatment (*P* < 0.05, Fig. 5b and c), which indicates that P2X7 receptors are required for the enhancement by shock waves of MTX-induced apoptosis. In addition, to verify the key role for P2X7 receptor in the cell membrane permeability, we determined the uptake of Calcein and LY following addition of KN62 and found significantly reduced intake of Calcein and LY following addition of KN62 (Fig. 6). These results suggest that shock wave treatment promoted intake of MTX by altering cell membrane permeability via P2X7 receptor-ATP signaling, thereby enhancing methotrexate-induced apoptosis.

## Discussion

**Fig. 7:**
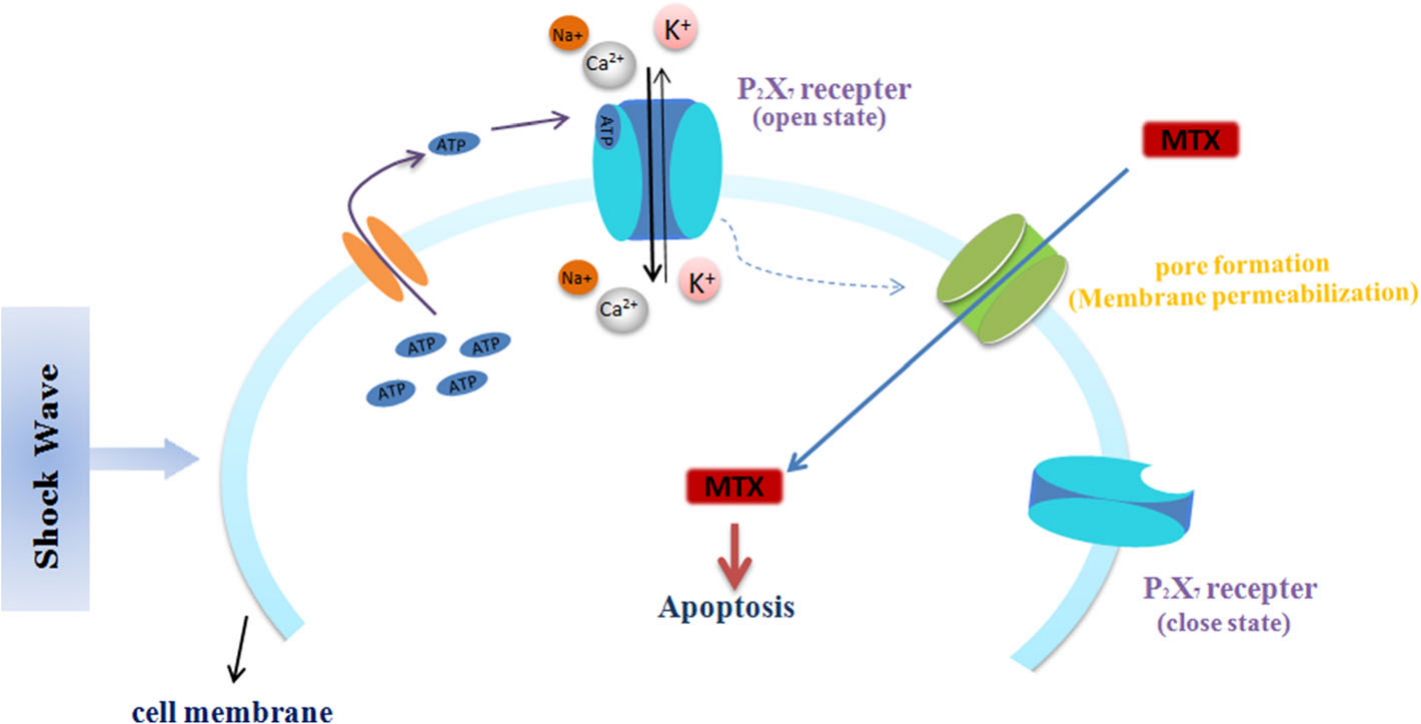
The proposed mechanism by which shockwave treatment increases osteosarcoma cell death: Shockwave promote the release of intracellular ATP, which in turn binds P2X7 receptors that contribute to the opening of cell membrane channels that allow entrance of MTX into cells,by which the osteosarcoma cell death increases

In this study, we demonstrated that subjecting cells to fewer than moderate shock wave treatment does not cause a significant loss of U2OS cell viability. We found viability of U2OS cells remained >95 % following <450 shock wave pulses at 7 kV or <200 pulses at 14 kV, which were used for further experiments. Shock waves significantly promoted the uptake of fluorophores Calcein and LY. Moreover, better effects were obtained by adding fluorophore prior to shock waves, suggesting the shock wave can increase transient cell membrane permeability. More importantly, we also found that shock waves significantly enhanced the uptake of MTX and the cytotoxicity of cells to this chemotherapeutic drug. This finding was consistent with the results by Palmero [8], however, in their study, the underlying mechanism was not investigated. We further found that P2X7 was highly expressed on the surface of U2OS cells and that shock waves increased the extracellular concentration of ATP. In addition, we found that ATP had a significant effect on cell viability with concentrations ≥100 μM only, while the highest ATP efflux concentration by shock wave was 1.6 μM, indicating that ATP and P2X7 receptors do not directly induce apoptosis following shock waves. Our data showed that shock wave treatment enhanced MTX-induced apoptosis through mechanisms that involve cell membrane permeability changes mediated by ATP release and P2X7 receptor stimulation. The proposed mechanism by which shockwave treatment increases osteosarcoma cell death is depicted in Fig. 7: shock wave cells induce efflux of intracellular ATP, which binds P2X7 receptors to increase membrane permeability and influx of MTX into the cell, ultimately leading to apoptosis. Our findings suggest that shock wave treatment could represent a promising adjuvant therapy for osteosarcoma.

Since its discovery, shock wave therapy has been widely used in the clinical treatment of various diseases including kidney stones, obesity [14], cancer [15], and burns [16]. Recently, shock waves have been used to treat tendinitis, femoral head necrosis, and nonunion fractures [17]. The aim of this work was to explore whether shock waves can be used as an adjuvant therapy for osteosarcoma. First, we investigated the shock wave conditions that must be maintained to reduce overwhelming cell disruption. We found that the majority of cells could withstand shock wave treatments with <450 pulses at 7 kV or <200 pulses at 14 kV. Similar results were obtained in MC3T3 cells.

Gambihler et al. previously reported that shock waves could permeabilize plasma membrane of L1210 mouse leukemia cells [18]. Using two fluorophores, Calcein and LY, we investigated the effect of shock waves on the permeability of U2OS osteosarcoma cells. We found that shock wave treatment significantly increased the uptake of these dyes when compared to control cells that take up a small quantity of fluorophore likely by fluid-phase pinocytosis or other mechanisms [19]. In addition we found that cells treated with higher shock wave intensity absorbed more fluorophore than cells treated with lower intensity. Based on these results, we hypothesized that shock wave treatment would also increase the uptake of MTX. Indeed, we found that shock wave treatment significantly increased intracellular concentrations of MTX and enhanced the cytotoxicity of MTX on U2OS osteosarcoma cells.

Shock waves have been previously reported to stimulate the release of ATP into the extracellular compartment by accelerating the fusion of ATP-rich vesicles with the cell membrane [13] or by opening of cell membrane channels [20–23]. By application of GdCl_3_ to block channels and BFA to inhibit ATP production, shock waves still elevated ATP levels, suggesting there exists the third mechanism that the high-strength instant positive phase pressure generated by shock wave during a few seconds may cause a transient increase of cell membrane permeability, leading to a burst of ATP outflow. Our results demonstrate that shock wave-induced release of ATP did not contribute to promotion of cell death. Further experiments indicated that shock waves significantly enhanced MTX-induced apoptosis, which was however significantly reduced by inhibition of P2X7 receptors. Our data suggest that release of ATP and binding of ATP to P2X7 receptors contributes to the opening of cell membrane channels to facilitate uptake of MTX by cells, resulting in subsequent apoptosis. We thus speculate that shock waves promote the release of intracellular ATP, which in turn binds P2X7 receptors that contribute to the opening of cell membrane channels that allow entrance of macromolecules into cells (Fig. 7).

## Conclusions

In conclusion, shock waves promote U2OS cell uptake of the chemotherapeutic drug MTX, and enhances MTX-induced cytotoxicity. Shock waves induce ATP release and enhance MTX-induced apoptosis in a P2X7 receptor-dependent manner. Our findings suggest that shock wave treatment may be a potential adjuvant therapy for the control of osteosarcoma. Therefore, after chemotherapy, osteosarcoma patients may be treated by local tumor shock wave therapy to promote the uptake of chemotherapy drugs by osteosarcoma cells. This strategy may reduce the dosage of chemotherapy drugs, alleviate side effects of chemotherapy drugs, but also improve the treatment outcome of osteosarcoma.

## Abbreviations

BFA: Brefeldin A
DMSO: Dimethylsulfoxide
LY: Lucifer Yellow CH dilithium salt
MTT: 3-(4, 5-dimethylthiazol-2-yl)-2, 5-diphenyltetrazolium bromide
MTX: Methotrexate
OD: Optical density
RPMI: Roswell Park Memorial Institute
